# Diagnostic accuracy of three ultrasonography strategies for deep vein thrombosis of the lower extremity: A systematic review and meta-analysis

**DOI:** 10.1371/journal.pone.0228788

**Published:** 2020-02-11

**Authors:** Noémie Kraaijpoel, Marc Carrier, Grégoire Le Gal, Matthew D. F. McInnes, Jean-Paul Salameh, Trevor A. McGrath, Nick van Es, David Moher, Harry R. Büller, Patrick M. Bossuyt, Mariska M. G. Leeflang

**Affiliations:** 1 Department of Vascular Medicine, Amsterdam UMC, University of Amsterdam, Amsterdam Cardiovascular Sciences, Amsterdam, the Netherlands; 2 Department of Medicine, Division of Hematology, The Ottawa Hospital, Ottawa, ON, Canada; 3 Ottawa Hospital Research Institute, Ottawa, ON, Canada; 4 Faculty of Medicine, University of Ottawa, Ottawa, ON, Canada; 5 Clinical Epidemiology Program, Ottawa Hospital Research Institute, The Ottawa Hospital, ON, Canada; 6 Department of Radiology, University of Ottawa, Ottawa, ON, Canada; 7 School of Epidemiology, Public Health and Preventive Medicine, Ottawa University, Ottawa, ON, Canada; 8 Department of Clinical Epidemiology, Biostatistics, and Bioinformatics, Amsterdam UMC, University of Amsterdam, Amsterdam, the Netherlands; Institut d'Investigacions Biomediques de Barcelona, SPAIN

## Abstract

**Background:**

Compression ultrasonography (CUS) is the first-line imaging test in the diagnostic management of suspected deep vein thrombosis (DVT) of the lower extremity. Three CUS strategies are used in clinical practice. However, their relative diagnostic accuracy is uncertain.

**Objectives:**

This systematic review and meta-analysis aimed to summarize and compare the diagnostic accuracy of single limited, serial limited, and whole-leg CUS for DVT.

**Methods:**

MEDLINE, Embase, and CENTRAL were searched from January 1^st^, 1989 to July 23^rd^, 2019 for studies assessing at least one of the CUS strategies in adults with suspected DVT of the lower extremity, using clinical follow-up for venous thromboembolism or contrast venography as the reference standard. Study selection, data extraction, and risk of bias assessment were performed in duplicate by independent authors. A bivariate random-effects model was used to compute diagnostic accuracy summary estimates.

**Results:**

Forty studies (n = 21,250) were included. The venous thromboembolic event rate after a negative CUS (failure rate) of single limited (1.4%; 95% CI, 0.83–2.5), serial limited (1.9%; 95% CI, 1.4–2.5), and whole-leg CUS (1.0%; 95% CI, 0.6–1.6) did not differ significantly. The proportion of positive results was lower with single limited CUS, as was DVT prevalence in this group.

**Conclusions:**

The failure rates of single limited, serial limited, and whole-leg CUS for DVT appeared to be quite comparable. The relative failure rate of single limited CUS remains uncertain, as the DVT prevalence was lower in these studies. Therefore, this CUS strategy may only be safe in a selected group of low-risk patients. Preference for one of the strategies may be based on pretest probability assessment, feasibility, expertise, and perceived clinical relevance of isolated distal DVT.

## Introduction

The first-line imaging test in the diagnostic management of patients presenting with clinically suspected deep vein thrombosis (DVT) is compression ultrasonography (CUS). Historically, contrast venography was the gold standard for DVT diagnosis, which assessed both distal and proximal deep veins of the lower extremity. When CUS emerged, its diagnostic accuracy for distal DVT was found to be suboptimal when compared with venography. However, technology improvements have led to better visualization of the deep venous system with CUS and at present, three CUS strategies are interchangeably used in clinical practice: single limited, serial limited, and whole-leg CUS.

Limited CUS, also called two-point, rapid, or proximal CUS, is easier and faster to perform as only the proximal deep veins of the lower extremity (i.e. popliteal veins or more proximal vessels) are assessed. It can be performed in a single or serial approach. The latter includes a second CUS examination after 5 to 10 days following an initial negative CUS to evaluate if a possible distal DVT has propagated to the proximal veins.

Whole-leg CUS, also referred to as complete CUS, is a single examination of the distal and proximal deep veins of the lower extremity, thereby detecting both distal and proximal DVT. It is relatively time-consuming and technically more demanding than limited CUS. Therefore, availability may differ between centers, depending on expertise and feasibility at busy emergency departments.

The relative diagnostic performance of the different CUS strategies is unclear. Current guidelines include conflicting recommendations with regard to preferred CUS strategy [1–5]. This guidance is mostly based on indirect comparisons between the strategies, as intra-individual comparisons and randomized trials in this particular field are scarce. The present systematic review of published literature and a meta-analysis of the reported results aimed to summarize and compare the diagnostic accuracy of single limited, serial limited, and whole-leg CUS for DVT.

## Methods

This systematic review followed the reporting recommendations of the Preferred Reporting Items for Systematic reviews and Meta-Analyses for Diagnostic Test Accuracy statement (PRISMA-DTA checklist; **S1 Appendix**) [6]. The protocol was registered at the International Prospective Register of Systematic Reviews Registry (PROSPERO; CRD42018086651).

### Literature search

A systematic search was conducted on July 23^rd^, 2019, in MEDLINE and Embase from January 1^st^, 1989 (the year in which the first high-quality study evaluating two-point CUS for suspected DVT was published [7]) up to the search date, combining terms for ‘deep vein thrombosis’ and ‘ultrasonography’ (see **S2 Appendix** for search strategies). The Cochrane Central Register of Controlled Trials (CENTRAL) was searched from April 1^st^, 2019 to July 23^rd^, 2019. In addition, conference proceedings of the American Society of Hematology (2004 to 2018) and the International Society for Thrombosis and Hemostasis (2003 to 2019), and references in reports of eligible studies were hand searched. The search was restricted to original studies reported in English, German, Dutch, French, Italian, or Spanish.

### Eligibility criteria

Studies evaluating single or serial limited and/or single whole-leg CUS in adults in whom DVT of the lower extremity was clinically suspected in the inpatient or outpatient setting were eligible. At least one ultrasonography strategy had to be evaluated in all-comers or in a subgroup of patients referred for imaging based on pre-test probability assessment and/or D-dimer testing.

Limited CUS was defined as either a two-point or (extended) proximal approach. The two-point technique includes an examination of two venous segments, i.e. the common femoral vein at the level of the inguinal ligament and the popliteal vein in the popliteal fossa [8]. The (extended) proximal strategy examines additional segments of the proximal venous system, and may include the common and superficial femoral veins, the popliteal vein, and sometimes includes the confluence of the deep calf veins (i.e. calf trifurcation). Limited CUS comprises either a single or a serial examination in which a second assessment is performed after 5 to 10 days.

Whole-leg CUS was defined as an examination of both the proximal and distal deep venous system of the leg, including the femoral veins, the popliteal vein, the posterior and anterior tibial vein, and the peroneal vein. It may include the muscular veins (gastrocnemius or soleus).

Two types of studies were included: 1) contemporary diagnostic management studies in which only patients with a negative ultrasonography were assessed with a reference standard consisting of a clinical follow-up of at least 45 days for the occurrence of venous thromboembolism (i.e. DVT and fatal or non-fatal pulmonary embolism), and 2) earlier diagnostic accuracy studies in which contrast venography was the reference method.

Studies were excluded if patients were younger than 18 years, if those with a negative CUS or venography were systematically treated with anticoagulants, if diagnostic accuracy measures could not be obtained or reconstructed, or if different reference standards were used following CUS in the subgroups of interest.

### Study selection, data extraction, and risk of bias and applicability assessment

Three authors (NK, JPS, and TMG) independently screened titles, abstracts, and subsequently full-text articles for eligibility. In case of disagreement between the reviewers, a discussion was held to reach consensus. Data extraction was performed by three independent authors (NK, JPS, and TMG) using standardized piloted forms, including study characteristics, patient characteristics, index and reference test characteristics, additional ultrasonography modalities, 2 x 2 data (total number of positives, true positives, false positives, true negatives, and false negatives), and venous thromboembolic events and mortality during follow-up. In case only a subgroup underwent the index test, only data from that specific subgroup were extracted. Diagnosis of DVT at baseline or venous thromboembolism during follow-up was considered confirmed as per the study physician’s judgement based on objective imaging.

Summary estimates were used to reconstruct 2 x 2 data in case such tables were not reported. Inconclusive or non-diagnostic test results were excluded from the 2 x 2 tables and were documented separately.

The risk of bias and applicability of each included study was independently assessed by three authors (NK, JPS, and TMG) using the QUADAS-2 (Quality Assessment of Diagnostic Accuracy Studies) tool [9], of which the items were adjusted as appropriate for the present study.

### Outcomes of interest

Diagnostic accuracy measures of single limited, serial limited, and whole-leg CUS were separately assessed for 1) diagnostic management studies using clinical follow-up to document the occurrence of venous thromboembolic events, and 2) earlier diagnostic accuracy studies that had used contrast venography as the clinical reference standard.

For studies using clinical follow-up, the failure rate was the primary outcome, which is the proportion of patients with a negative ultrasonography at baseline who were diagnosed with a venous thromboembolic event during follow-up. In these studies, the lack of a reference test at baseline limits assessment of true and false positive test results, as all positive CUS examinations are considered diagnostic for DVT. As a consequence, evaluation of the sensitivity, specificity, and positive predictive value is hampered. Therefore, to provide information on the ratio between positive and negative test results, the proportion of positive results was also assessed. This measure is highly correlated with the prevalence of DVT among the examined patients.

For studies in which contrast venography was used as the reference standard, the false negative rate (1-negative predictive value) was the primary outcome. Estimates of sensitivity, specificity, positive predictive value, and negative predictive value were also assessed.

### Statistical analysis

For the primary analysis, only studies with a low risk of bias and no concerns regarding applicability based on QUADAS-2 evaluation were included. Diagnostic accuracy measures were computed from the 2 x 2 contingency tables for each individual study.

A bivariate logit-normal random-effects model was used to compute diagnostic accuracy summary estimates with 95% confidence intervals (CI). Diagnostic accuracy summary estimates of the different CUS strategies were compared by adding CUS strategy type as covariate to the bivariate model.

Statistical heterogeneity was evaluated by estimating the between-study variance (tau-squared) and by providing a 95% prediction interval (PI), which is an estimate of the interval in which 95% of future observations in similar studies will fall. Potential sources of heterogeneity were explored using random-effects meta-regression analyses in which different study or patient characteristics were added to the model as dichotomous and/or continuous covariates, including DVT prevalence, patient selection prior to CUS examination (all-comers versus selected patients based on pre-test probability assessment and/or D-dimer), duration of symptoms, publication date, and proximal (examination of the proximal veins starting at the popliteal vein) versus extended limited ultrasonography (examination of the proximal veins starting at the calf trifurcation). Several additional pre-planned subgroup analyses were hampered by a lack of studies or unpublished data.

Additionally, sensitivity analyses were performed in which all studies were included, regardless of QUADAS-2 appraisal.

Statistical tests were two-sided and P-values below 0.05 were regarded as indicating statistical significance. All analyses were performed in R (R Foundation for Statistical Computing, Vienna, Austria, https://www.R-project.org), using the ‘mada’ package for the bivariate logit-normal random effects model and for meta-regression analyses. Forest plots were designed with Review Manager (RevMan version 5.3; Copenhagen: The Nordic Cochrane Centre, The Cochrane Collaboration, 2014).

### Role of the funding source

This project was supported by The Netherlands Organization for Scientific Research (NWO; project number 015.012.052). The funder had no role in the design and conduct of the study, or in the decision to submit the manuscript.

## Results

### Study characteristics

**Fig 1:**
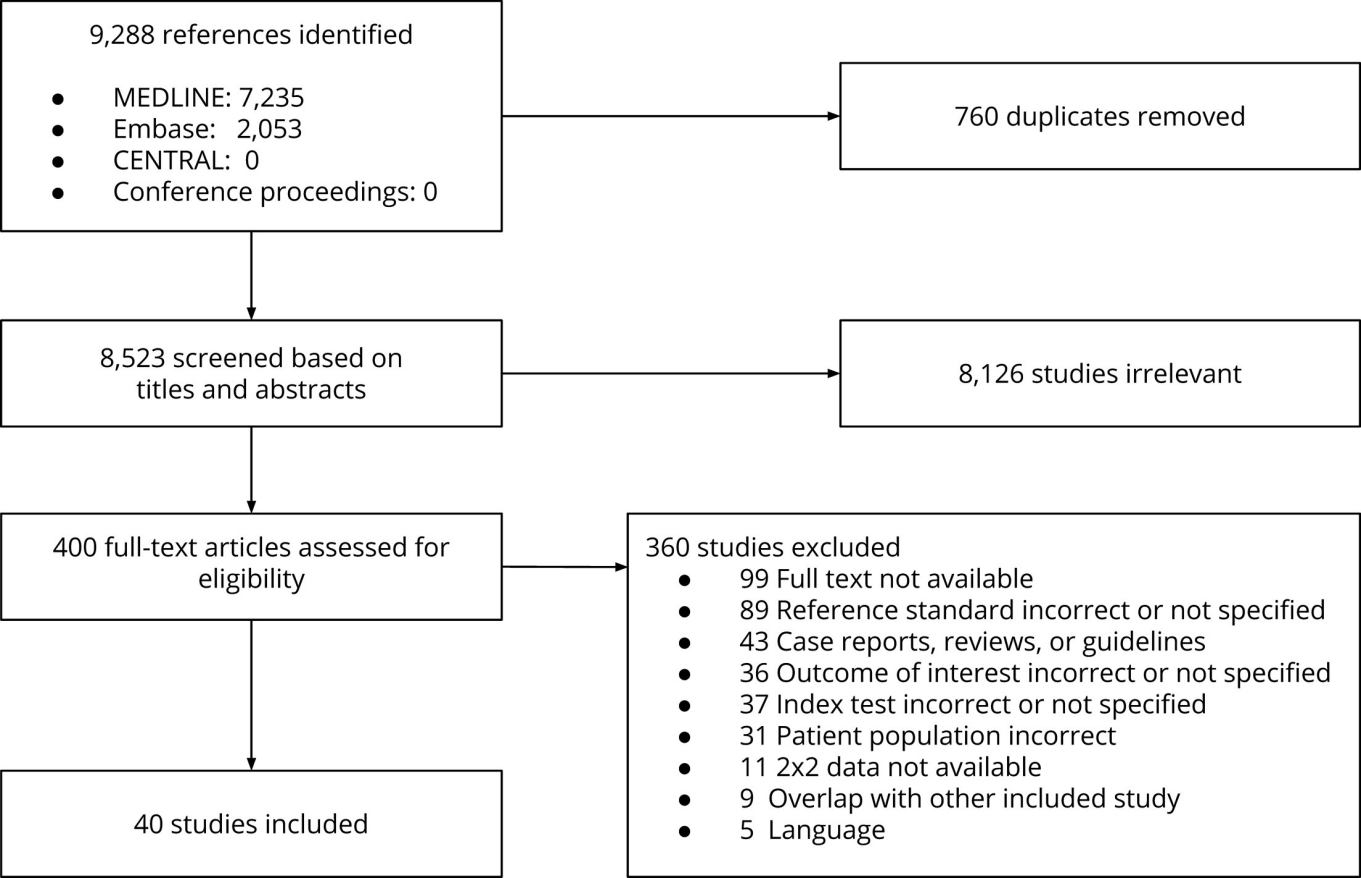
PRISMA flowchart.

Of the 9,288 citations identified in the literature search, 40 studies (n = 21,250) were eligible and included in the analyses (see **Fig 1** for the PRISMA flowchart) [7,10–48]. Twenty-seven studies (68%), published between 1991 and 2017 (median, 2003), used clinical follow-up for venous thromboembolic events as reference standard. The remaining 13 studies used contrast venography (median year of publication, 1991; range, 1989 to 2007).

Twenty-two studies (55%; n = 15,779) were considered to have a low risk of bias and no concerns regarding applicability (**S3 Appendix**). The remaining 18 studies showed risk of bias or applicability concerns in at least one domain; mostly in the patient selection domain (n = 13; 33%), as it was often unclear whether a consecutive or random sample was enrolled (n = 10; 25%). In addition, in studies with contrast venography as the reference standard, knowledge of the reference standard results at interpretation of the index test (n = 3; 23%) and the time frame between CUS and venography assessment (n = 3; 23%) were often unclear.

Characteristics of the included studies are detailed in **S4 Appendix**. Thirty-six (90%) were prospective studies and four had a retrospective design. One study was in the primary care setting, the remaining 39 (98%) in the secondary care setting, of which 6 were reported to be in the emergency department setting only. Sample size ranged from 38 to 1,739 patients.

Characteristics of the index and comparator test and of the reference standard are shown in **S5 Appendix.** None of the studies had directly compared CUS strategies intra-individually. However, one study randomized patients to undergo either serial limited or whole-leg CUS [25]. Single limited CUS was evaluated in 16 studies (40%), serial limited CUS in 10 (25%), and single whole-leg CUS in 19 (48%). In studies with clinical follow-up as the reference standard, follow-up duration was 3 months, except for one study in which it was 6 months [35]. Ultrasonography was reported to be performed by radiologists in 10 studies (25%), ultrasonography technicians in 9 (23%), vascular physicians in 6 (15%).

Patient characteristics are detailed in **S6 Appendix.** Mean age ranged from 29 to 72 years (median, 60), the proportion of males from 0 to 68% (median, 41%), and the DVT prevalence from 2.8 to 59% (median, 25%).

### Diagnostic accuracy in studies using clinical follow-up as reference standard

#### Single limited compression ultrasonography

**Fig 2:**
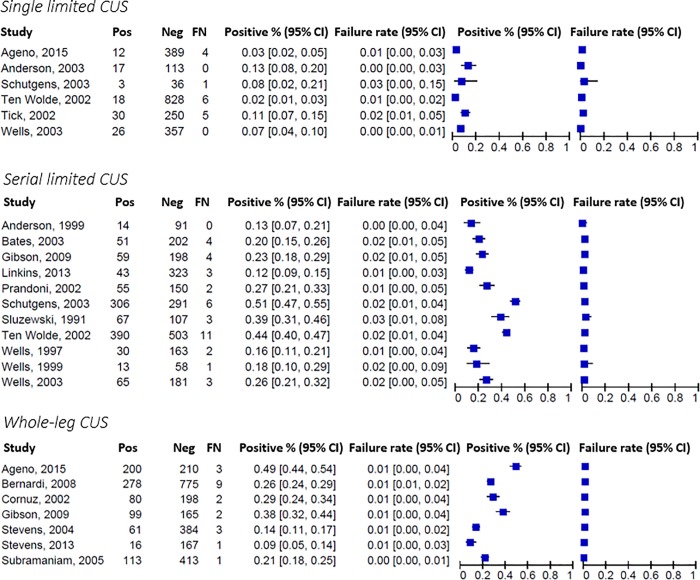
Forest plots diagnostic accuracy of compression ultrasonography in studies that used clinical follow‐up as a reference standard. Abbreviations: CI: confidence interval, CUS: compression ultrasonography. *Pos* and *Neg* indicate the number of positive and negative CUS results; *FN* (false negative) indicates the number of venous thromboembolic events during follow-up in those with a negative CUS result.

Six studies assessed single limited CUS of which 2,079 patients could be included in the meta-analysis [10,12,38,43,44,47]. Before CUS examination, patients were managed with the use of different diagnostic algorithms and were selected for imaging based on pretest probability assessment and/or D-dimer testing (**S7 Appendix**). Median DVT prevalence was 8.5% (IQR, 4.7 to 12). Summary estimates were 1.4% (95% CI, 0.83 to 2.5; 95% PI, 0.42 to 4.8) for the failure rate and 6.4% (95% CI, 3.5 to 11; 95% PI, 0.84 to 35) for the proportion of positive results (see **Fig 2** for forest plots; see **Table 1** for summary estimates). There was evidence of substantial heterogeneity across the results of the included studies, which was associated with DVT prevalence (as a continuous variable: p<0.001 for the proportion of positive results, p = 0.13 for the failure rate in meta-regression analysis).

**Table 1:** Summary estimates diagnostic accuracy of compression ultrasonography in studies that used clinical follow-up as a reference standard.

Ultrasonography technique	Studies, n	Patients, n	DVT prevalence, median (IQR)	Proportion of positive results (95% CI; 95% PI)	τ2*	Failure rate† (95% CI; 95% PI)	τ2*
Single limited CUS	6	2,079	8.5% (4.7–12)	6.4% (3.5–11; 0.84–35)	0.63	1.4% (0.83–2.5; 0.42–4.8)	0.54
Serial limited CUS	11	3,360	25% (18–34)	25% (18–33; 6.8–60)	0.60	1.9% (1.4–2.5; 1.1–3.2)	0.51
Whole-leg CUS	7	3,159	27% (18–34)	25% (16–36; 4.4–70)	0.64	1.0% (0.6–1.6; 0.37–2.5)	0.52

Abbreviations: CI: confidence interval, CUS: compression ultrasonography, PI: prediction interval

* Tau-squared (τ^2^) represents the between-study variance and indicates the degree of heterogeneity.

†The failure rate is the proportion of patients with a negative ultrasonography at baseline who were diagnosed with venous thromboembolism during follow-up.

Subgroup analyses were hampered by a lack of studies per group (**S8 Appendix**). Sensitivity analyses including all studies regardless of quality showed comparable results (n = 9 [10–12,19,33,38,43,44,47]; see **S9 Appendix** for summary estimates).

#### Serial limited compression ultrasonography

Serial limited CUS was evaluated in 11 studies of which 3,360 patients were included in the meta-analysis [12,15,25,31,35,38,39,43,45–47]. Patients were either all-comers or were selected for imaging based on pretest probability assessment and/or D-dimer testing (**S7 Appendix**). Median DVT prevalence was 25% (IQR, 18 to 34). Median proportion of DVTs detected at second examination was 5.1% (IQR, 3.2 to 7.1). The summary failure rate was 1.9% (95% CI, 1.4 to 2.5; 95% PI, 1.1 to 3.2) and the proportion of positive results was 25% (95% CI, 18 to 33; 95% PI 6.8 to 60; see **Fig 2** for forest plots; see **Table 1** for summary estimates). Heterogeneity was partly explained by DVT prevalence (as a continuous variable: p<0.001 for the proportion of positive results, p = 0.23 for the failure rate in meta-regression analysis).

Subgroup analyses are shown in **S8 Appendix**. Diagnostic accuracy was comparable between all-comers and patients referred for imaging guided by pre-test probability and/or D-dimer, and was similar for proximal and extended limited CUS.

Sensitivity analyses including all studies regardless of quality included the same studies.

#### Whole-leg compression ultrasonography

Seven studies assessed whole-leg CUS of which 3,159 patients were included in the meta-analysis [10,17,22,25,40–42]. Patients were either all-comers or were selected for imaging based on pretest probability assessment and/or D-dimer testing (**S7 Appendix**). Median DVT prevalence was 27% (IQR, 18 to 34). The proportion of isolated distal DVTs relative to the total number of diagnosed DVTs ranged between 23 and 62% (**S10 Appendix**). The failure rate was 1.0% (95% CI, 0.6 to 1.6; 95% PI, 0.37 to 2.5) and the pooled proportion of positive results was 25% (95% CI, 16 to 36; 95% PI, 4.4 to 70; see **Fig 2** for forest plots; see **Table 1** for summary estimates). Heterogeneity was partly explained by DVT prevalence (as a continuous variable: p<0.001 for the proportion of positive results, p = 0.277 for the failure rate in meta-regression analysis).

Subgroup analyses are shown in **S8 Appendix**. Diagnostic accuracy was comparable between all-comers and patients referred for imaging based on pre-test probability and/or D-dimer. Sensitivity analyses including all studies regardless of quality showed comparable results (n = 12 [10,17,21–25,29,30,40–42]; see **S9 Appendix** for summary estimates).

#### Comparison compression ultrasonography strategies

The failure rate did not differ significantly between the three techniques (serial limited vs. single limited CUS: p = 0.36 for meta-regression; whole-leg vs. single limited CUS: p = 0.51, and serial limited vs. whole-leg CUS: p = 0.08).

The proportion of positive results was significantly higher with serial limited and whole-leg CUS compared with single limited CUS (p<0.001 and p<0.001, respectively), and was comparable between serial and whole-leg CUS (p = 0.95).

### Diagnostic accuracy in studies using contrast venography as reference standard

#### Single limited compression ultrasonography

**Fig 3:**
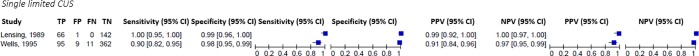
Forest plots diagnostic accuracy of compression ultrasonography in studies that used contrast venography as a reference standard. Abbreviations: CI: confidence interval, CUS: compression ultrasonography, FN: false negative, FP: false positive, NPV: negative predictive value, PPV: positive predictive value, TN: true negative, TP: true positive.

Single limited CUS was assessed in 2 studies totaling 686 patients of whom all underwent CUS [7,48]. DVT prevalence in these studies was 22% and 32%, respectively. Summary estimates for the detection of proximal DVT were 1.3% (95% CI, 0.1 to 10; 95% PI, 0.0 to 100) for the false negative rate, 96% (95% CI, 64 to 100; 95% PI, 0.0 to 100) for sensitivity, 98% (95% CI, 95 to 99; 95% PI, 0.0 to 100) for specificity, 95% (95% CI, 81 to 99; 95% PI, 0.0 to 100) for the positive predictive value, and 99% (95% CI, 90 to 100; 95% PI, 0.0 to 100) for the negative predictive value (see **Fig 3** for forest plots and **Table 2** for summary estimates).

**Table 2:** Summary estimates diagnostic accuracy of compression ultrasonography in studies that used contrast venography as a reference standard.

Ultrasonography technique	Studies, n	Patients, n	DVT prevalence	Sensitivity (95% CI; 95% PI)	τ2*	Specificity (95% CI; 95% PI)	τ2*	PPV (95% CI; 95% PI)	τ2*	NPV (95% CI; 95% PI)	τ2*	False negative rate† (95% CI; 95% PI)
Single limited CUS‡	2	686	22% and 32%	96% (64–100; 0–100)	0.95	98% (95–99; 0–100)	0.58	95% (81–99;0–100)	0.71	99% (90–100; 0–100)	0.88	1.3% (0.1–10; 0–100)
Whole-leg CUS§	1	75	45%	79% (62–91; NR)	-	88% (73–95; NR)	-	84% (66–94; NR)	-	84% (69–93; NR)	-	16% (7.4–31; NR)

Abbreviations: CI: confidence interval, CUS: compression ultrasonography, DVT: deep vein thrombosis, NPV: negative predictive value, NR: not reported, PI: prediction interval, PPV: positive predictive value

* Tau-squared (τ^2^) represents the between-study variance and indicates the degree of heterogeneity.

† The failure rate equals 1-NPV

‡ Diagnostic accuracy for proximal DVT

§ Raw data from a single study by Rose et al. published in 1990 [37].

Sensitivity analyses including all studies regardless of quality showed comparable results (n = 6 [7,20,26,32,36,48]; see **S11 Appendix** for summary estimates).

#### Serial limited compression ultrasonography

None of the studies in which contrast venography was the reference standard assessed serial limited CUS.

#### Whole-leg compression ultrasonography

One study published in 1990 (n = 75) assessed whole-leg CUS in all patients [37]. DVT prevalence was 45%. The proportions of proximal and isolated distal DVTs were not reported. The false negative rate was 16% (95% CI, 7.4 to 31), sensitivity was 79% (95% CI, 62 to 91), specificity 88% (95% CI, 73 to 95), positive predictive value 84% (95% CI, 66 to 94), and negative predictive value 84% (95% CI, 69 to 93).

Sensitivity analyses including all studies regardless of quality showed comparable results (n = 7 [14,16,18,27,28,34,37]; see **S11 Appendix** for summary estimates).

#### Comparison compression ultrasonography strategies

The diagnostic accuracy of the three techniques across studies that had used contrast venography as reference test could not be compared due to a lack of studies in both groups.

## Discussion

This systematic review summarizes the diagnostic accuracy of single limited, serial limited, and whole-leg CUS for the diagnosis of DVT. In earlier studies with contrast venography as the reference method, whole-leg CUS appeared to have a higher false negative rate than single limited CUS (16% vs. 1.3%). However, in more recent studies using clinical follow-up as the reference method, the thromboembolic event rate during follow-up (failure rate) appeared to be comparable among the three techniques. The proportion of positive test results was significantly lower with single limited CUS (6.4%) compared with serial limited and whole-leg CUS (both 25%), which is mainly explained by the difference in DVT prevalence among the groups (median prevalence, 8.5% vs. 25% and 27%, respectively). Likely, pretest selection with clinical decision rules and D-dimer caused the difference in DVT prevalence among the three groups.

Single limited, serial limited, and whole-leg CUS are the current imaging strategies for the diagnosis of DVT. Preference for one strategy over the other differs between centers and ultrasonographers. Recent guidelines and consensus statements have had conflicting recommendations regarding the preferred strategy. The 2018 American Society of Hematology, the 2012 American College of Chest Physicians, and the 2012 National Institute for Health and Care Excellence guidelines recommend single limited CUS for patients deemed to have a low pretest probability of DVT and serial limited or whole-leg CUS for those with a moderate or high probability [1–3]. Two recent consensus reports published in 2018 recommend either whole-leg US [4] or no preference for a particular CUS strategy [5], regardless of pretest probability. However, recommendations are mostly based on the efficiency and safety of the individual strategies and expert opinion, as evidence on direct comparisons between the three strategies is scarce.

In the present meta-analysis, more recent studies using clinical follow-up as the reference method showed very comparable failure rates of the three strategies. This could imply that there may not be a preferred strategy when taking only safety into account. Importantly, the failure rate is highly dependent on DVT prevalence. In studies assessing single limited CUS, DVT prevalence was lower, most likely due to pretest selection of lower-risk patients with clinical decision rules and/or D-dimer. Although speculative, a higher prevalence in the single limited CUS group may have led to a higher failure rate. Therefore, the safety of single limited CUS relative to the other strategies remains unclear.

In the earlier studies, in which the reference method was contrast venography, the false negative rate was higher with whole-leg CUS than with single limited CUS (16% vs. 1.3%, respectively). Although ultrasonography technology may have been outdated compared with modern ultrasonography standards, these results suggest that visualization of the distally located, smaller veins as assessed by whole-leg CUS is suboptimal. Theoretically, based on these findings, 16% of patients with a negative whole-leg examination would be expected to develop a thromboembolic event during follow-up. However, as previously discussed, in the studies using clinical follow-up as the reference standard, the thromboembolic event rate during follow-up of whole-leg CUS was comparable with that of the other strategies (1.0 to 1.9%). Otherwise stated, although whole-leg CUS misses one in six thrombi as detected by contrast venography, in the studies with clinical follow-up the thromboembolic event rate after a negative whole-leg CUS appears to be low. This leads to the hypothesis that the majority of thrombi missed by whole-leg CUS are self-limiting and do not progress to symptomatic thromboembolic events during follow-up.

Whole-leg CUS showed isolated distal DVT in 23 to 62% of all detected DVT cases. Previous studies have questioned the clinical relevance of distal DVT and anticoagulant treatment for this condition remains controversial [49]. The CACTUS trial, which is the only randomized placebo-controlled trial to date, compared 6-week nadroparin therapy with placebo in patients with isolated distal DVT who were considered to have a low risk of recurrent venous thromboembolism [50]. The results of this study suggest that anticoagulant treatment in patients with isolated distal DVT may cause more harm than benefit as the primary outcome rate (extension of calf DVT to proximal veins, contralateral proximal DVT, and symptomatic pulmonary embolism) was comparable between both groups (3.3% vs. 5.4%; p = 0.54) at the expense of a higher rate of clinically relevant bleeding in the nadroparin group (4% vs. 0%; p = 0.0255). However, the results of the study should be interpreted with caution, since it was prematurely terminated when only half of the estimated sample size was included. The 2016 American College of Chest Physicians guideline suggests that anticoagulant treatment should not be given to all patients with isolated distal DVT [51]. However, at present no validated tools are available to discriminate between low- and high-risk patients and to guide decisions on anticoagulant therapy in these patients. The clinical relevance and anticoagulant treatment strategies of distal DVT will further determine the role of whole-leg CUS in the diagnostic management of DVT.

Strengths of this study include the complete overview of diagnostic accuracy studies of the three CUS strategies for DVT and the use of a bivariate meta-analysis model, which incorporates any correlation that might exist between two diagnostic accuracy measures. Only studies with a low risk of bias and low concerns regarding applicability were included in the primary analysis, which increases the validity of the findings. Studies or subgroups of patients in which different reference standards were used were excluded from the analysis, thereby preventing differential verification bias.

Several limitations deserve to be acknowledged. There was substantial heterogeneity across the included studies, most likely due to differences in DVT prevalence, but other factors such as experience of the ultrasonographers, ultrasonography technology at the time of the study, and use of additional ultrasonography modalities may also have contributed. The prevalence of DVT varied widely across the patients assessed with CUS. Yet, as DVT prevalence in clinical practice may also differ depending on the use of diagnostic algorithms including pretest probability assessment and D-dimer testing, geographical location, and patient comorbidity, the findings of the present study were considered to apply to current practice. None of the included studies directly compared CUS techniques intra-individually. However, the only randomized study included in the present analysis found similar results as the overall analysis. In total, 521 patients with an abnormal D-dimer or a ‘DVT likely’ Wells score were randomized to undergo serial limited or whole-leg CUS; the failure rates were found to be comparable (2.0% [95% CI, 0.6 to 5.1] vs. 1.2% [95% CI, 0.2 to 4.3]; p = 0.69, respectively) [25]. Several important subgroup analyses, such as for patients with suspected recurrent DVT, could not be performed due to a lack of studies per subgroup or unreported data. In the main analysis both patients with and without a history of venous thromboembolism were included. Moreover, the effect of different modalities on the diagnostic accuracy of ultrasonography could not be studied. Language restrictions for study selection may have led to undetected studies. However, we expect that the vast majority of studies performed on this subject have been published in the languages deemed eligible, as also reflected in the low number of studies excluded based on language at full-text screening (n = 5).

The results from the present study suggest that single limited, serial limited, and whole-leg CUS may be considered equivalent in clinical practice regarding safety. Several factors may be considered when choosing for one of the strategies. From a practical point of view, a single diagnostic examination may be preferred over a serial approach as the latter implies that the patient must return for a second examination when the first examination was negative for DVT. However, performing whole-leg CUS may not always be feasible, for example at the emergency department with limited time to examine the patient or when CUS is performed at the bedside by less experienced ultrasonographers.

In conclusion, the failure rates of single limited, serial limited, and whole-leg CUS for DVT were found to be quite comparable in patients selected as per the individual study diagnostic algorithms. The relative safety of single limited CUS remains uncertain as the DVT prevalence was lower in the included studies. Therefore, use of this CUS strategy in higher prevalence groups may not be appropriate. Preference for one of the strategies should be based on pretest probability assessment, feasibility, and expertise. Future studies should focus on direct comparisons of the diagnostic accuracy, for example by randomizing patients with suspected DVT who are referred for imaging to each of the three CUS strategies. The clinical relevance of isolated distal DVT found by whole-leg CUS as well as the need for anticoagulant treatment should also be further assessed.

## Supporting information

S1 Appendix PRISMA-DTA checklist. (DOCX)

S2 Appendix Search strategies. (DOCX)

S3 Appendix Results risk of bias and applicability concerns assessment according to the QUADAS-2 tool. (DOCX)

S4 Appendix Study characteristics. (DOCX)

S5 Appendix Index and comparator test, and reference standard characteristics. Abbreviations: CUS: compression ultrasonography, DVT: deep vein thrombosis, PE: pulmonary embolism, US: ultrasonography *Limited CUS is restricted to the proximal deep veins of the lower extremity and can be categorized into (1) two-point CUS, which assesses the common femoral and popliteal veins; (2) three-point CUS, which assesses the common femoral and popliteal vein, and the calf trifurcation; (3) proximal CUS, which assesses any of the proximal deep veins of the lower extremity starting at the popliteal vein; and (4) extended proximal CUS, which assesses any of the proximal deep veins of the lower extremity starting at the calf trifurcation. Limited CUS is performed as a single examination (single limited CUS) or is repeated after 5 to 10 days in case of a negative result (serial limited CUS). Whole-leg CUS assesses both proximal and distal deep veins of the lower extremity.†Only a subgroup of patients was included in the meta-analysis.
(DOCX)

S6 Appendix Patient characteristics. Abbreviations: CUS: compression ultrasonography, DVT: deep vein thrombosis, PE: pulmonary embolism * DVT prevalence in the subgroup of patients that was included in the meta-analysis.
(DOCX)

S7 Appendix Selection of patients prior to ultrasonography examination. Abbreviations: CUS: compression ultrasonography PTP: pretest probability Patients were all-comers or were selected for CUS imaging with the use of a diagnostic algorithm consisting of a pretest probability (PTP) assessment and/or D-dimer. PTP was classified as either low, moderate, or high, or as ‘DVT likely’ or ‘DVT unlikely’.
(DOCX)

S8 Appendix Subgroup analyses—Summary estimates diagnostic accuracy of compression ultrasonography in studies that used clinical follow-up as a reference standard. Abbreviations: CI: confidence interval, CUS: compression ultrasonography, DVT: deep vein thrombosis, PTP: pretest probability assessment *Patients undergoing CUS were either all-comers or were referred for imaging based on pretest probability assessment and/or D-dimer testing †Proximal limited CUS includes examination of the popliteal vein up to the femoral vein, extended limited CUS also includes the calf trifurcation. ‡P-value for meta-regressionSubgroup analyses for age, body mass index, history of venous thromboembolism, duration of symptoms, ultrasonography modalities, ultrasonography operator, and retrospective versus prospective study design were hampered as the number of studies in several subgroups was lower than 2.
(DOCX)

S9 Appendix Sensitivity analysis including all studies regardless of quality—Summary estimates diagnostic accuracy of compression ultrasonography in studies that used clinical follow-up as a reference standard. Abbreviations: CI: confidence interval, CUS: compression ultrasonography, DVT: deep vein thrombosis, PI: prediction interval * Tau-squared (τ^2^) represents the between-study variance and indicates the degree of heterogeneity. †The failure rate is the proportion of patients with a negative ultrasonography at baseline who were diagnosed with venous thromboembolism during follow-up.
(DOCX)

S10 Appendix Distribution of proximal and distal deep vein thrombosis diagnosed with whole-leg compression ultrasonography in studies that used clinical follow-up as a reference standard. Abbreviations: DVT: deep vein thrombosis.
(DOCX)

S11 Appendix Sensitivity analysis including all studies regardless of quality—Summary estimates diagnostic accuracy of compression ultrasonography in studies that used contrast venography as a reference standard. Abbreviations: CI: confidence interval, CUS: compression ultrasonography, DVT: deep vein thrombosis, NPV: negative predictive value, PI: prediction interval, PPV: positive predictive value * Tau-squared (τ^2^) represents the between-study variance and indicates the degree of heterogeneity. †The false negative rate equals 1-NPV.
(DOCX)

S12 Appendix Dataset. (7Z)
