# Supplementary material for: Diagnostic accuracy of three ultrasonography strategies for deep vein thrombosis of the lower extremity: A systematic review and meta-analysis
Source: PLoS One. 2020 Feb 11;15(2):e0228788. doi: 10.1371/journal.pone.0228788 (PMC7012434; doi:10.1371/journal.pone.0228788)
Supplement: S2 Appendix — (DOCX) [file pone.0228788.s002.docx]

**S2 Appendix. Search strategies**

**Search date:** July 23^rd^, 2019

**MEDLINE**

**1.** “Ultrasonography”[MeSH Terms:NoExp] OR ultrason*[tiab] OR ultrasound*[tiab] OR echograph*[tiab] OR sonogr*[tiab] OR duplex[tiab] OR doppler[tiab]

**2.** “Venous thrombosis”[MeSH Terms:NoExp] OR “Venous thromboembolism”[MeSH Terms:NoExp] OR (deep AND (vein[tiab] OR venous[tiab]) AND (thrombos*[tiab] OR thrombi*[tiab] OR thrombus[tiab] OR thromboembol*[tiab])) OR deep-vein[tiab] OR deep-venous[tiab]

**3.** “1989/01/01”[pdat]:”2019/07/23”[pdat]

**4.**   1. AND 2. AND  3.

n = 7,235

**Embase**

**1.** ultrasound/ or echography/ or ultrason*.ti,ab. or ultrasound*.ti,ab. or echograph*.ti,ab. or sonogr*.ti,ab. or duplex.ti,ab. or doppler.ti,ab.

**2.** vein thrombosis/ or venous thromboembolism/ or deep vein thrombosis/ or deep venous thrombosis/ or deep venous thrombus/ or vein thrombos*,ti.ab. or venous thrombos*.ti,ab. or venous thromboembolism*.ti,ab. or deep-vein.ti,ab. or deep-venous.ti,ab.

**3.** 1. and 2.

**4.** limit 3 to yr = “1989-Current”, and exclude MEDLINE journals

n = 2,053
