# Supplementary material for: Diagnostic accuracy of three ultrasonography strategies for deep vein thrombosis of the lower extremity: A systematic review and meta-analysis
Source: PLoS One. 2020 Feb 11;15(2):e0228788. doi: 10.1371/journal.pone.0228788 (PMC7012434; doi:10.1371/journal.pone.0228788)

**S3 Appendix. Results risk of bias and applicability concerns assessment according to the QUADAS-2 tool**
**1. Risk of bias and applicability concerns per study**

| **Study** | **Risk of bias** | **Applicability concerns** |
| --- | --- | --- |
| *Reference standard: clinical follow-up* | | |
| Ageno, 2015 | Low risk of bias | Low concern regarding applicability |
| Aguilar, 2007 | Risk of bias | Low concern regarding applicability |
| Anderson, 2003 | Low risk of bias | Low concern regarding applicability |
| Anderson, 1999 | Low risk of bias | Low concern regarding applicability |
| Bates, 2003 | Low risk of bias | Low concern regarding applicability |
| Bernardi, 2008 | Low risk of bias | Low concern regarding applicability |
| Chan, 2013 | Low risk of bias | Concerns regarding applicability |
| Cornuz, 1999 | Risk of bias | Concerns regarding applicability |
| Cornuz, 2002 | Low risk of bias | Low concern regarding applicability |
| Dybowska, 2015 | Risk of bias | Concerns regarding applicability |
| Elias, 2003 | Risk of bias | Low concern regarding applicability |
| Gibson, 2009 | Low risk of bias | Low concern regarding applicability |
| Le Gal, 2006 | Low risk of bias | Concerns regarding applicability |
| Le Gal, 2012 | Low risk of bias | Concerns regarding applicability |
| Linkins, 2013 | Low risk of bias | Low concern regarding applicability |
| Mitsunaga, 2017 | Risk of bias | Low concern regarding applicability |
| Prandoni, 2002 | Low risk of bias | Low concern regarding applicability |
| Schutgens, 2003 | Low risk of bias | Low concern regarding applicability |
| Sluzewski, 1991 | Low risk of bias | Low concern regarding applicability |
| Stevens, 2004 | Low risk of bias | Low concern regarding applicability |
| Stevens, 2013 | Low risk of bias | Low concern regarding applicability |
| Subramaniam, 2005 | Low risk of bias | Low concern regarding applicability |
| Ten Wolde, 2002 | Low risk of bias | Low concern regarding applicability |
| Tick, 2002 | Low risk of bias | Low concern regarding applicability |
| Wells, 1997 | Low risk of bias | Low concern regarding applicability |
| Wells, 1999 | Low risk of bias | Low concern regarding applicability |
| Wells, 2003 | Low risk of bias | Low concern regarding applicability |

| *Reference standard: contrast venography* | | |
| --- | --- | --- |
| Aywak, 2007 | Risk of bias | Concerns regarding applicability |
| Baxter, 1990 | Risk of bias | Concerns regarding applicability |
| Cavaye, 1990 | Risk of bias | Concerns regarding applicability |
| Chance, 1991 | Risk of bias | Concerns regarding applicability |
| Gundmundsen, 1990 | Risk of bias | Concerns regarding applicability |
| Heijboer, 1992 | Risk of bias | Low concern regarding applicability |
| Kennedy, 1999 | Risk of bias | Concerns regarding applicability |
| Lensing, 1989 | Low risk of bias | Low concern regarding applicability |
| Mantoni, 1989 | Risk of bias | Concerns regarding applicability |
| Pasquariello, 1999 | Risk of bias | Concerns regarding applicability |
| Quintavalla, 1992 | Risk of bias | Low concern regarding applicability |
| Rose, 1990 | Low risk of bias | Low concern regarding applicability |
| Wells, 1995 | Low risk of bias | Low concern regarding applicability |

**2. Studies in which clinical follow-up was the reference standard**

**2a. Risk of bias assessment**


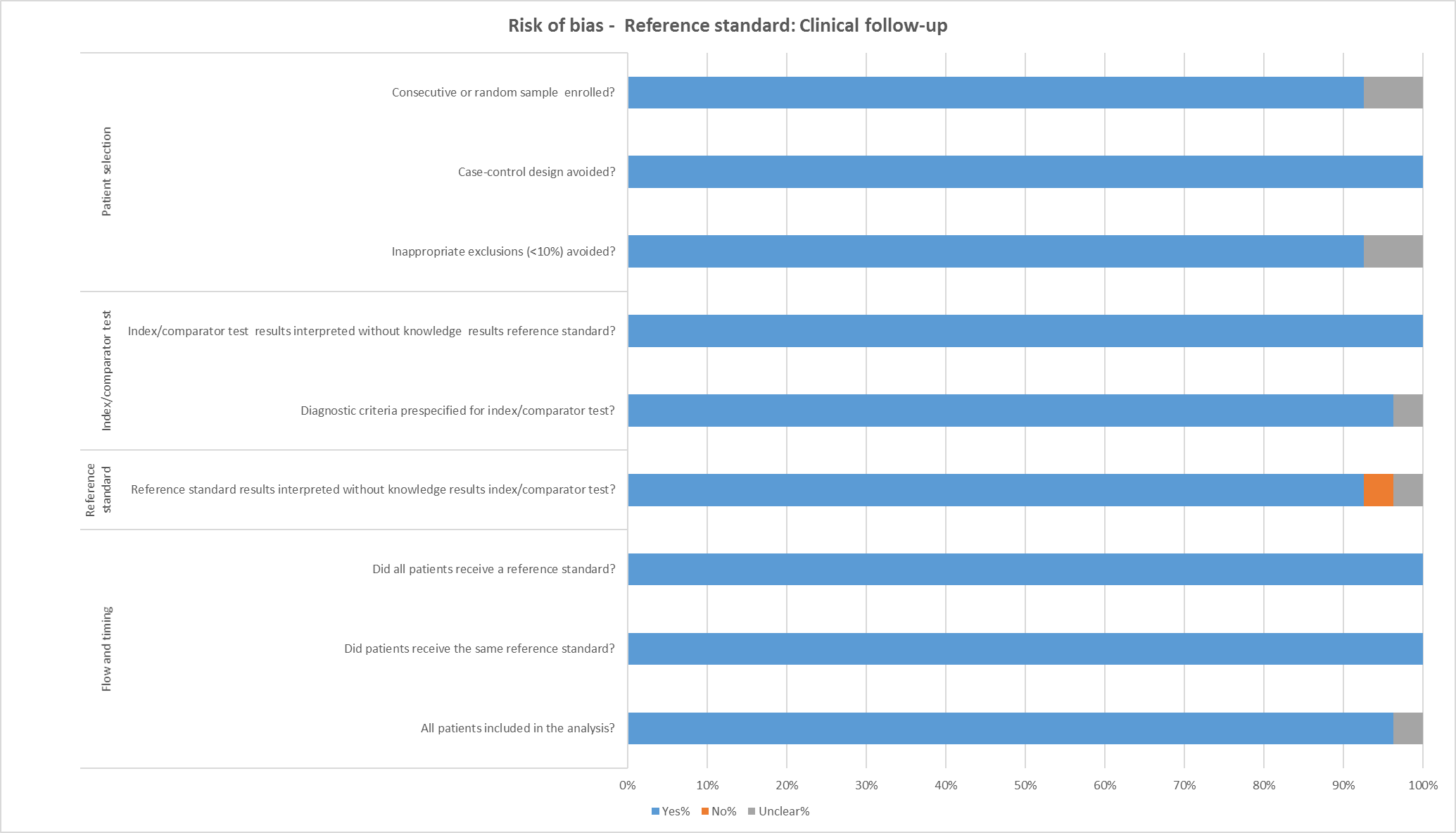


**2b. Risk of bias assessment per study domain**

**2c. Applicability concerns assessment**

**
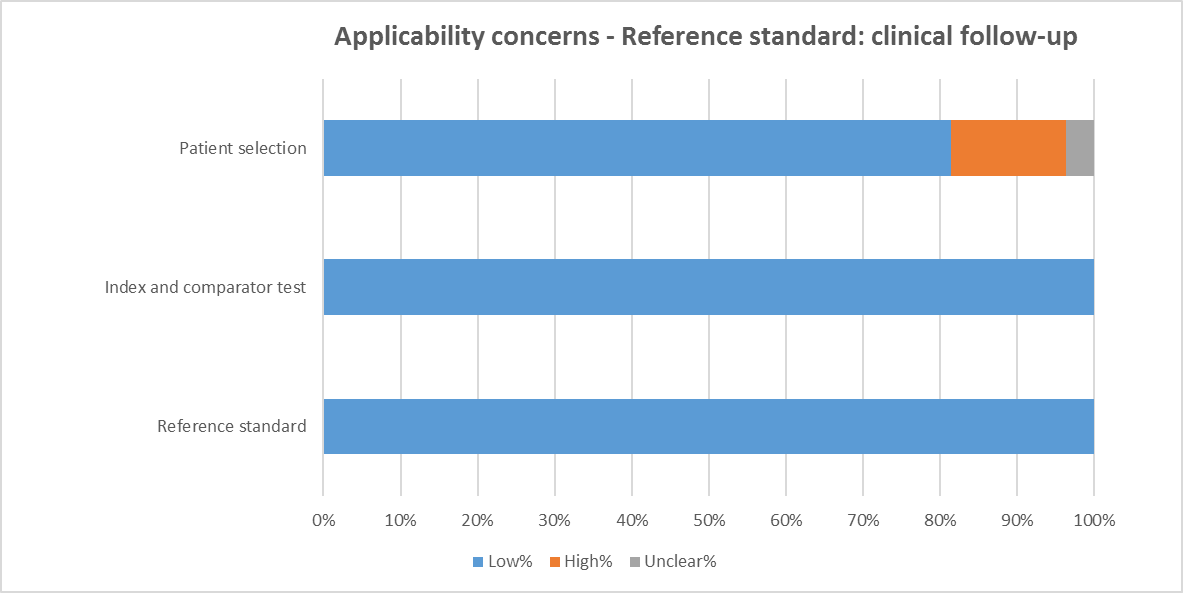
**

**3. Studies in which contrast venography was the reference standard**

**3a. Risk of bias assessment**


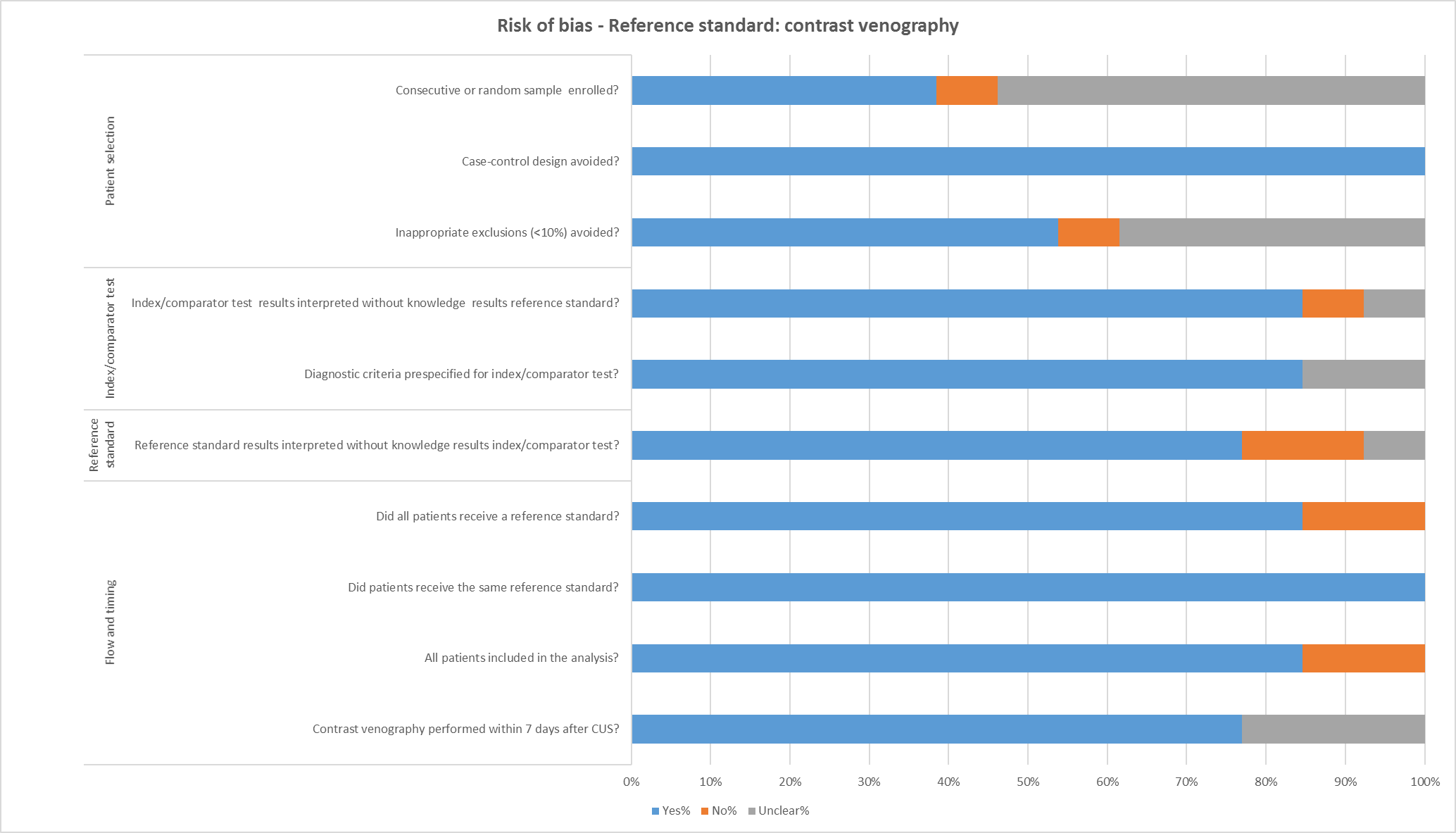


**3b. Risk of bias per study domain**


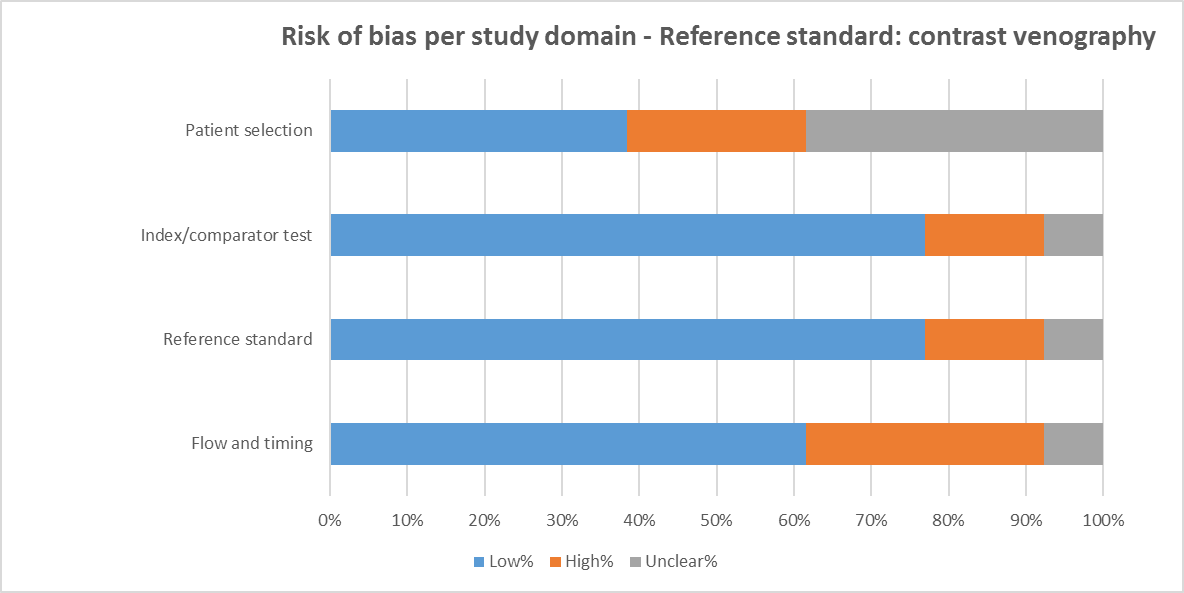


**3c. Applicability concerns assessment**


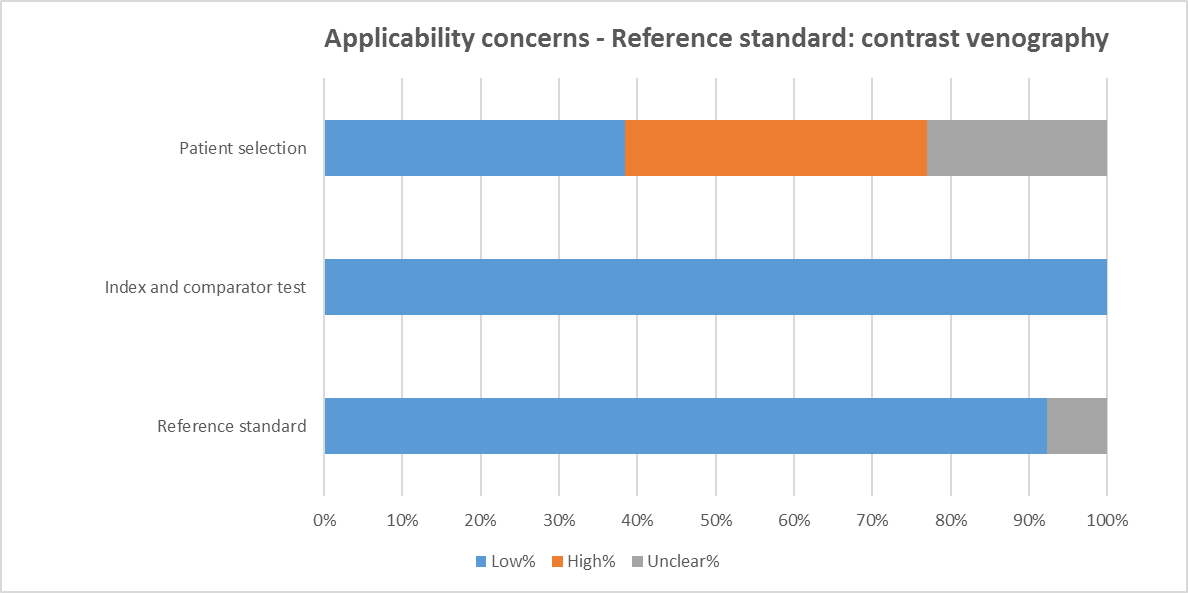

Supplement: S3 Appendix — (DOCX) [file pone.0228788.s003.docx]
