# Supplementary material for: Diagnostic accuracy of three ultrasonography strategies for deep vein thrombosis of the lower extremity: A systematic review and meta-analysis
Source: PLoS One. 2020 Feb 11;15(2):e0228788. doi: 10.1371/journal.pone.0228788 (PMC7012434; doi:10.1371/journal.pone.0228788)
Supplement: S4 Appendix — (DOCX) [file pone.0228788.s004.docx]

**S4 Appendix. Study characteristics**

| **Study** | **Aim** | **Design** | **Clinical care setting** | **Inclusion period** | **Geographical area** | **Sample size, n** | **Funding sources** |
| --- | --- | --- | --- | --- | --- | --- | --- |
| **Ageno, 2015** [10] | To assess the diagnostic value of an algorithm combining whole-leg and limited compression ultrasonography for suspected DVT. | Prospective | Secondary | 2011-2014 | Belgium, Canada, Italy, the Netherlands, and Switzerland | 1,162 | - |
| **Aguilar, 2007** [11] | To evaluate the diagnostic value of D-Dimer testing and the safety of a diagnostic strategy consisting of a pretest probability assessment, D-dimer testing, and ultrasonography for suspected DVT. | Prospective | Secondary | 2004-2005 | Spain | 105 | - |
| **Anderson, 2003** [12] | To determine the safety of algorithms combining pretest probability assessment and D-dimer testing for suspected DVT in the emergency department setting. | Prospective | Secondary | 1997-1999 | Canada | 1075 | - |
| **Anderson, 1999** [13] | To evaluate the performance of a diagnostic algorithm consisting of a clinical model and ultrasonography for suspected DVT. | Prospective | Secondary | 1994-1996 | Canada | 347 | - |
| **Aywak, 2007** [14] | To compare the findings of venous sonography with contrast venography for suspected DVT. | Prospective | Secondary | 2002-2003 | Kenya | 44/55 limbs | - |
| **Bates, 2003** [15] | To determine whether a negative result on a quantitative latex D-dimer assay eliminates the need for further investigation in patients with a low or moderate pretest probability of DVT. | Prospective | Secondary | 1999-2001 | Canada | 556 | Canadian Institutes of Health Research and bioMérieux Inc. |
| **Baxter, 1990** [16] | To evaluate colour Doppler ultrasonography in the diagnostic management of suspected DVT. | Prospective | Secondary | - | UK | 40 | - |
| **Bernardi, 2008** [17] | To assess two diagnostic strategies in the management of symptomatic outpatients with suspected DVT of the lower extremities. | Prospective | Secondary | 2003-2006 | Italy | 2,098 | Società Italiana per lo Studio dell’Emostasi e della Trombosi and AGEN Biomedical Ltd (Brisbane, Australia) |
| **Cavaye, 1990** [18] | To report on the experience and results of lower limb duplex scans in the investigation of DVT. | Retrospective | Secondary | 1988-1989 | Australia | 53/56 limbs | - |
| **Chan, 2013** [19] | To evaluate the diagnostic accuracy of serial compression ultrasonography and Doppler imaging of the proximal veins over a 7-day period among symptomatic pregnant women for suspected DVT. | Prospective | Secondary | 2002-2010 | Canada | 221 | Heart and Stroke Ontario |
| **Chance, 1991** [20] | To assess the role of ultrasound in the emergency department diagnosis of DVT. | Prospective | Secondary | - | USA | 70 | - |
| **Cornuz, 1999** [21] | To determine the diagnostic value of whole-leg ultrasonography in symptomatic patients suspected of having DVT. | Retrospective | Secondary | - | USA, Switzerland | 997 | Swiss National Science Foundation |
| **Cornuz, 2002** [22] | To compare implicit clinical assessment and the Wells score, in isolation and in combination with rapid quantitative D-dimer measurements for suspected DVT. | Prospective | Secondary | - | Switzerland | 278 | - |
| **Dybowska, 2015** [23] | To evaluate the diagnostic accuracy of the Wells score for suspected DVT in a primary care setting. | Prospective | Primary | 2007-2009 | Poland | 1,048 | - |
| **Elias, 2003** [24] | To prospectively evaluate the clinical outcomes of patients after a single negative complete ultrasonography for suspected DVT. | Prospective | Secondary | - | France | 623 | - |
| **Gibson, 2009** [25] | To compare the safety and feasibility of rapid and complete compression ultrasonography for suspected DVT. | Prospective | Secondary | 2002-2007 | Germany, Australia, the Netherlands | 1,002 | - |
| **Gudmundsen, 1990** [26] | To assess the accuracy of ultrasonography for suspected DVT using venography as the gold standard. | Prospective | Secondary | 1988-1989 | Norway | 150 | - |
| **Heijboer, 1992** [27] | To evaluate the accuracy of impedence plethysmography and real¬time compression ultrasonography for the detection of DVT. | Prospective | Secondary | - | Netherlands, Italy | 83 | Trombosestichting Nederland |
| **Kennedy, 1999** [28] | To assess the accuracy of duplex ultrasonography for suspected DVT performed by sonographers with little experience of the technique. | Prospective | Secondary | 1997-1998 | UK | 38 | - |
| **Le Gal, 2006** [30] | To assess the safety of a single complete compression ultrasonography to rule out the diagnosis of DVT in pregnant or early postpartum women. | Retrospective | Secondary | 2002-2004 | France | 162 | - |
| **Le Gal, 2012** [29] | To assess the safety of ruling out the diagnosis of DVT in pregnant or early postpartum women using single compression ultrasonography. | Prospective | Secondary | 2006-2009 | France, Switzerland | 210 | Projet Hospitalier de Recherche Clinique, Swiss National Foundation |
| **Lensing, 1989** [7] | To evaluate the diagnostic accuracy of real-time B-mode ultrasonography for suspected DVT with vein compressibility used as the sole criterion . | Prospective | Secondary | 1987-1988 | Netherlands, Italy | 225 | Netherlands Heart Foundation |
| **Linkins, 2013** [31] | To evaluate a diagnostic algorithm for suspected DVT using different D-dimer thresholds depending on pretest probability assessment. | Prospective | Secondary | 2004-2010 | Canada | 1,723 | Heart and Stroke Foundation of Ontario |
| **Mantoni, 1989** [32] | To assess the accuracy of duplex sonography in diagnosing DVT. | Prospective | Secondary | - | Denmark | 90 | - |
| **Mitsunaga, 2017** [33] | To determine the three month rate of symptomatic VTE and clinical outcomes of inpatients and ambulatory patients with suspected DVT after an initially normal single proximal ultrasonography result. | Retrospective | Secondary | 2014 (jan-dec) | Hawaii | 1,295 | - |
| **Pasquariello, 1999** [34] | The assess the diagnostic accuracy of ultrasonography in the diagnosis of DVT. | Prospective | Secondary | - | Sweden | 77 | - |
| **Prandoni, 2002** [35] | To assess the safety of withholding anticoagulant therapy from patients with suspected recurrent ipsilateral DVT who had improved or stable vein diameter assessments during serial (day 1, 2 and 7) testing. | Prospective | Secondary | 1992-1999 | Netherlands, Italy | 205 | - |
| **Quintavalla, 1992** [36] | To evaluate the accuracy of Duplex ultrasonography for suspected DVT. | Prospective | Secondary | 1988-1989 | Italy | 165 | - |
| **Rose, 1990** [37] | To evaluate the accuracy and technical limitations of color duplex flow imaging in patients with suspected DVT. | Prospective | Secondary | - | USA | 69/75 limbs | - |
| **Schutgens, 2003** [38] | To evaluate the safety of the combination of a non-high pretest clinical probability score and a normal D-dimer level to replace ultrasonography as the initial test in the diagnostic management of patients with suspected DVT. | Prospective | Secondary | - | Netherlands | 827 | - |
| **Sluzewski, 1991** [39] | To evaluate the safety of withholding anticoagulants in symptomatic patients with suspected DVT who have repeated negative ultrasonography tests. | Prospective | Secondary | 1988-1989 | Netherlands | 174 | - |
| **Stevens, 2004** [40] | To determine whether negative results on a single examination with comprehensive duplex ultrasonography are adequately sensitive to justify withholding anticoagulation in patients with suspected DVT. | Prospective | Secondary | 2000-2001 | USA | 445 | By the Deseret Foundation, Salt Lake City, Utah (grant no. 371). |
| **Stevens, 2013** [41] | To evaluate the rate of VTE in patients with a ‘DVT likely’ pretest probability and a single negative whole-leg ultrasonography. | Prospective | Secondary | 2005-2010 | USA | 183 | Intermountain Research and Medical Foundation, Murray, Utah |
| **Subramaniam, 2005** [42] | To determine the accuracy of negative findings of a complete lower limb ultrasonography examination to justify withholding anticoagulation therapy | Prospective | Secondary | 2001-2003 | New Zealand, Australia | 526 | - |
| **Ten Wolde, 2002** [43] | To assess the diagnostic accuracy of D-dimer testing in cancer patients with suspected DVT. | Prospective | Secondary | 1995-1999 | Netherlands, Italy | 1,739 | - |
| **Tick, 2002** [44] | To evaluate the safety of withholding anticoagulant treatment in patients with suspected DVT who have a low clinical probability test and a normal ultrasonography. | Prospective | Secondary | 1997-2000 | Netherlands | 811 | - |
| **Wells, 1997** [45] | To validate a diagnostic algorithm including assessment with a clinical model and ultrasonography for suspected DVT. | Prospective | Secondary | 1994-1996 | Canada | 593 | Physician Services Incorporated Foundation, the Heart and Stroke Foundation of Nova Scotia, Canada. |
| **Wells, 1999** [46] | To determine if the use of the clinical model in combination with ultrasound could be used to provide safe and feasible management of hospitalized patients with suspected deep-vein thrombosis. | Prospective | Secondary | 1994-1996 | Canada | 150 | Physician Services Incorporated Foundation and the Heart and Stroke Foundation of Nova Scotia. |
| **Wells, 2003** [47] | To evaluate the efficacy and safety of a diagnostic algorithm consisting of D-dimer and ultrasonography. | Prospective | Secondary | - | Canada | 1,096 | Supported by the Heart and Stroke Foundations of Ontario and Nova Scotia, Canada |
| **Wells, 1995** [48] | To compare the accuracy of impedance plethysmography and compression ultrasonography to venography. | Prospective | Secondary | - | Canada, Italy | 495 | McLaughlin Scholarship University of Ottawa |
