# Supplementary material for: Diagnostic accuracy of three ultrasonography strategies for deep vein thrombosis of the lower extremity: A systematic review and meta-analysis
Source: PLoS One. 2020 Feb 11;15(2):e0228788. doi: 10.1371/journal.pone.0228788 (PMC7012434; doi:10.1371/journal.pone.0228788)
Supplement: S5 Appendix — Abbreviations: CUS: compression ultrasonography, DVT: deep vein thrombosis, PE: pulmonary embolism, US: ultrasonography *Limited CUS is restricted to the proximal deep veins of the lower extremity and can be categorized into (1) two-point CUS, which assesses the common femoral and popliteal veins; (2) three-point CUS, which assesses the common femoral and popliteal vein, and the calf trifurcation; (3) proximal CUS, which assesses any of the proximal deep veins of the lower extremity starting at the popliteal vein; and (4) extended proximal CUS, which assesses any of the proximal deep veins of the lower extremity starting at the calf trifurcation. Limited CUS is performed as a single examination (single limited CUS) or is repeated after 5 to 10 days in case of a negative result (serial limited CUS). Whole-leg CUS assesses both proximal and distal deep veins of the lower extremity.†Only a subgroup of patients was included in the meta-analysis. (DOCX) [file pone.0228788.s005.docx]

**S5 Appendix. Index and comparator test, and reference standard characteristics**

| **Study** | **Index/comparator test*** | **Diagnostic criteria index/comparator test** | **US modalities** | **Reference standard** | **Diagnostic criteria reference standard** | **US operator** |
| --- | --- | --- | --- | --- | --- | --- |
| **Ageno, 2015** | **Index:** Single limited (three-point) CUS  **Comparator:** Whole-leg CUS | **Index:** Noncompressibility common femoral vein, popliteal vein, or calf vein trifuration.  **Comparator:** Noncompressibility femoral bifurcation, femoral veins, great saphenous vein junction, popliteal vein, calf vein trifurcation, axial veins, and muscular veins. | - | 3-month clinical follow-up | Objectively confirmed DVT, non-fatal or fatal PE | Vascular physicians |
| **Aguilar, 2007†** | Single limited (extended proximal) CUS | Noncompressibility common femoral vein, superficial femoral vein, popliteal vein, or calf vein trifurcation. | - | 3-month clinical follow-up | Objectively confirmed DVT | Radiologists |
| **Anderson, 2003†** | Single limited (extended proximal) CUS | Noncompressibility or constant intraluminal filling defect seen within a deep vein on more than one view. The entire proximal deep vein system between the proximal common femoral vein and the calf vein trifurcation was evaluated. | - | 3-month clinical follow-up | Objectively confirmed proximal DVT and non-fatal and fatal PE | - |
| **Anderson, 1999†** | Serial limited (extended proximal) CUS | Noncompressibility. The entire proximal deep vein system between the proximal common femoral vein and the calf vein trifurcation was evaluated. | Triplex | 3-month clinical follow-up | Objectively confirmed proximal DVT and PE | - |
| **Aywak, 2007** | Whole-leg CUS | Noncompressibility of the common or superficial femoral veins, popliteal vein, proximal calf veins. | Gray scale, Duplex, Triplex | Venography | Intraluminal filling defect or abrupt termination of the opaque column. | Radiologists |
| **Bates, 2003†** | Serial limited (extended proximal) CUS | Noncompressibility of the common femoral vein or popliteal vein. | Triplex | 3-month clinical follow-up | Objectively confirmed proximal DVT and PE | - |
| **Baxter, 1990** | Whole-leg CUS | Noncompressibility of the common and superficial femoral veins, saphenofemoral junction, popliteal vein, and proximal calf veins. | Triplex | Venography | Intraluminal filling defect | Radiologists |
| **Bernardi, 2008†** | Whole-leg CUS | Noncompressibility of the common, superficial, or  deep femoral vein, popliteal vein, calf vein trifurcation, peroneal vein, posterior tibial vein, and the muscular veins | - | 3-month clinical follow-up | Objectively confirmed DVT, non-fatal or fatal PE | Vascular physicians |
| **Cavaye, 1990** | Whole-leg CUS | Noncompressibility of the common, superficial, or deep femoral vein, popliteal vein, calf vein trifurcation, peroneal vein, and the posterior tibial vein | Gray scale, Duplex | Venography | - | US technicians |
| **Chan, 2013** | Single limited (extended proximal) CUS | Noncompressibility of the femoral vein, popliteal vein, or calf trifurcation or absence of flow in the iliac vein. | Gray scale, Duplex | 3 months clinical follow-up up to at least 6 weeks after delivery | Objectively confirmed DVT or PE | US technicians |
| **Chance, 1991** | Single limited (two-point) CUS | Noncompressibility of the common femoral vein or popliteal vein. | - | Venography | - | Radiologists |
| **Cornuz, 1999** | Whole-leg CUS | Noncompressibility of the common femoral vein, popliteal vein, posterior tibial vein, or peroneal vein. | Gray scale, Triplex | 3-month clinical follow-up | Venous thromboembolism diagnosis | US technicians |
| **Cornuz, 2002** | Whole-leg CUS | Noncompressibility of the proximal and distal veins. | Duplex | 3-month clinical follow-up | Objectively confirmed DVT or PE | Vascular physicians |
| **Dybowska, 2015** | Whole-leg CUS | Noncompressibility of the common femoral vein, femoral vein, popliteal vein, peroneal vein, anterior tibial vein, and posterior tibial vein | - | 3-month clinical follow-up | Objectively confirmed DVT or PE | Radiologists |
| **Elias, 2003** | Whole-leg CUS | Noncompressibility and the presence of an intraluminal thrombus in the common, profundus, or superficial femoral vein, popliteal vein, posterior tibial vein, fibular vein, gastrocnemius vein, soleal vein. | Gray scale, Duplex | 3-month clinical follow-up | Objectively confirmed DVT or PE | Vascular physicians |
| **Gibson, 2009** | **Index:** Serial limited (three-point) CUS  **Comparator:** Whole-leg CUS | **Index:** Noncompressibility of the common femoral vein, popliteal vein, or calf vein trifurcation. **Comparator:** Noncompressibility of the femoral bifurcation, great saphenous vein junction, the profunda femoris, femoral vein, popliteal vein, calf vein trifurcation, the paired posterior tibial veins, the paired peroneal veins, the lesser saphenous vein junction, or the gastrocnemial and soleal sinusoids. | - | 3-month clinical follow-up | Objectively confirmed DVT or PE | US technicians |
| **Gudmundsen, 1990** | Single limited (two-point) CUS | Noncompressibility of the common femoral vein, superficial femoral vein, or popliteal vein | - | Venography | A constant filling defect in the deep venous system. | Radiologists |
| **Heijboer, 1992** | Whole-leg CUS | Noncompressibility of the common femoral vein, popliteal vein, calf vein trifurcation, proximal portions of the deep venous system. | - | Venography | A constant intraluminal filling defect on two or more projections or persistent nonfilling of a venous segment | US technicians |
| **Kennedy, 1999** | Whole-leg CUS | Noncompressibility of the deep proximal veins or calf veins | Duplex | Venography | - | US technicians |
| **Le Gal, 2012** | Whole-leg CUS | Noncompressibility of the common femoral vein, superficial femoral vein, popliteal vein, posterior tibial, or peroneal vein. Absence of flow in the iliac vein. | Gray scale, Duplex | 3-month clinical follow-up | Objectively confirmed DVT or PE | Vascular physicians |
| **Le Gal, 2006** | Whole-leg CUS | Noncompressibility of the common femoral vein, superficial femoral vein, popliteal vein, tibial posterior vein, peroneal vein, gastrocnemius veins, or soleal veins. Lack of flow in the iliac vein and inferior vena cava. | Gray scale, Duplex | 3-month clinical follow-up | Objectively confirmed DVT or PE | US technicians |
| **Lensing, 1989** | Single limited (two-point) CUS | Noncompressibility of the common femoral vein or popliteal vein. | Gray scale, Valsalva/resp | Venography | Intraluminal filling defect | US technicians |
| **Linkins, 2013†** | Serial limited (extended proximal) CUS | Noncompressibility of the deep veins between the common femoral vein to the calf trifurcation. | - | 3-month clinical follow-up | Objectively confirmed proximal DVT or PE | - |
| **Mantoni, 1989** | Single limited (extended proximal) CUS | Noncompressibility of the common femoral vein, superficial femoral vein, saphenous vein, popliteal vein, calf vein trifurcation. | Gray scale, Duplex | Venography | Intraluminal filling defect | Radiologists |
| **Mitsunaga, 2017** | Single limited (proximal) CUS | Noncompressibility of the ileofemoral junction, saphenofemoral junction, common femoral, superficial femoral, or popliteal veins. | Triplex | 3-month clinical follow-up | Objectively confirmed venous thromboembolism | - |
| **Pasquariello, 1999** | Whole-leg CUS | Noncompressibility of the femoral vein, popliteal vein, or calf veins. | Triplex | Venography | Contrast filling defect | Radiologists |
| **Prandoni, 2002** | Serial limited (two-point) CUS | Noncompressibility of the common femoral vein or popliteal vein. | - | 6-month clinical follow-up | Objectively confirmed DVT, non-fatal or fatal PE | - |
| **Quintavalla, 1992** | Single limited (proximal) CUS | Noncompressibility of the common femoral, superficial femoral, deep femoral, greater saphenous, or popliteal vein. | Gray scale, Duplex | Venography | Intraluminal filling defect | - |
| **Rose, 1990** | Whole-leg CUS | Noncompressibility of the common femoral, superficial femoral, deep femoral,  greater saphenous, popliteal, posterior tibial, anterior tibial, peroneal vein. | Triplex, Resp/Valsalva | Venography | Intraluminal filling defect | - |
| **Schutgens, 2003†** | **Index:** Single limited (three-point) CUS  **Comparator:** Serial limited (three-point) CUS | **Index and comparator:** Noncompressibility of the common femoral vein, popliteal vein, or calf vein trifurcation. | Gray scale | 3-month clinical follow-up | DVT or PE | - |
| **Sluzewski, 1991** | Serial limited (two-point) CUS | Noncompressibility of the common femoral vein or popliteal vein. | - | 3-month clinical follow-up | Proximal DVT or PE | - |
| **Stevens, 2004** | Whole-leg CUS | Noncompressibility of the deep veins between inguinal ligament and the malleolus. | Gray scale, Triplex | 3 months at least clinical follow-up | DVT, PE, paradoxical embolism, death from venous thromboembolism | Vascular physicians |
| **Stevens, 2013** | Whole-leg CUS | Noncompressibility of the deep veins between the most proximally identified deep vein and the malleolus. | - | 3-month clinical follow-up | Objectively confirmed DVT, non-fatal or fatal PE | US technicians |
| **Subramaniam, 2005** | Whole-leg CUS | Noncompressibility of the common femoral vein, superficial femoral vein, popliteal vein and calf vein trifurcation, and all three deep calf veins. | Gray scale, Triplex | 3-month clinical follow-up | Objectively confirmed DVT, non-fatal or fatal PE | Radiologists |
| **Ten Wolde, 2002** | **Index:** Single limited (three-point) CUS  **Comparator:** Serial limited (three-point) CUS | **Index and comparator:** Noncompressibility of the common femoral vein, popliteal vein, or calf vein trifurcation. | - | 3-month clinical follow-up | Venous thromboembolism | - |
| **Tick, 2002†** | Single limited (three-point) CUS | Noncompressibility of the common femoral vein, popliteal vein, or calf vein trifurcation. | Gray scale | 3-month clinical follow-up | Objectively confirmed DVT or PE | - |
| **Wells, 1997†** | Serial limited (extended proximal) CUS | Noncompressibility of the deep veins between the common femoral vein and calf vein trifurcation | Duplex, Triplex | 3-month clinical follow-up | DVT or PE | - |
| **Wells, 1999†** | Serial limited (extended proximal) CUS | Noncompressibility of the deep veins between the common femoral vein and calf vein trifurcation | - | 3-month clinical follow-up | DVT or PE | - |
| **Wells, 2003†** | **Index:** Single limited (extended proximal) CUS  **Comparator:** Serial limited (extended proximal) CUS | **Index and comparator:** Noncompressibility of the deep veins between the common femoral vein and calf vein trifurcation | - | 3-month clinical follow-up | Proximal DVT or PE | - |
| **Wells, 1995** | Single limited (three-point) CUS | Noncompressibility of the common femoral vein, popliteal vein, or calf vein trifurcation. | - | Venography | Intraluminal filling defect | Radiologists |

Abbreviations: CUS: compression ultrasonography, DVT: deep vein thrombosis, PE: pulmonary embolism, US: ultrasonography

*Limited CUS is restricted to the proximal deep veins of the lower extremity and can be categorized into (1) two-point CUS, which assesses the common femoral and popliteal veins; (2) three-point CUS, which assesses the common femoral and popliteal vein, and the calf trifurcation; (3) proximal CUS, which assesses any of the proximal deep veins of the lower extremity starting at the popliteal vein; and (4) extended proximal CUS, which assesses any of the proximal deep veins of the lower extremity starting at the calf trifurcation. Limited CUS is performed as a single examination (single limited CUS) or is repeated after 5 to 10 days in case of a negative result (serial limited CUS). Whole-leg CUS assesses both proximal and distal deep veins of the lower extremity.
†Only a subgroup of patients was included in the meta-analysis
