# Supplementary material for: Diagnostic accuracy of three ultrasonography strategies for deep vein thrombosis of the lower extremity: A systematic review and meta-analysis
Source: PLoS One. 2020 Feb 11;15(2):e0228788. doi: 10.1371/journal.pone.0228788 (PMC7012434; doi:10.1371/journal.pone.0228788)
Supplement: S6 Appendix — Abbreviations: CUS: compression ultrasonography, DVT: deep vein thrombosis, PE: pulmonary embolism * DVT prevalence in the subgroup of patients that was included in the meta-analysis. (DOCX) [file pone.0228788.s006.docx]

**S6 Appendix. Patient characteristics**

| **Study** | **Age, y, mean** | **Male , n (%)** | **History of DVT or PE, n (%)** | **Symptoms duration, d, mean** | **Outpatients, n (%)** | **DVT prevalence, %** |
| --- | --- | --- | --- | --- | --- | --- |
| **Ageno, 2015** (limited CUS: n = 401)  (whole-leg CUS: n = 410) | **limited CUS:**  69 **whole-leg CUS:**  72 | **limited CUS:**  158 **whole-leg CUS:**  170 | **limited CUS:**  14  **whole-leg CUS:**  28 | **limited CUS:**  4  **whole-leg CUS:**  4 | **limited CUS:**  401 (100)  **whole-leg CUS:**  410 (100) | **limited CUS:**  4.0  **whole-leg CUS:**  50 |
| **Aguilar, 2007** (n = 105) | 69 | 52 (50) | 105 (100) | - | 105 (100) | 29***** |
| **Anderson, 2003** (n =1,075) | 57 | 471 (44) | - | 6 | 1,075 (100) | 13***** |
| **Anderson, 1999** (n = 347) | 54 | 154 (44) | - | 7 | 347 (100) | 13***** |
| **Aywak, 2007** (n = 44) | - | 30 (68) | - | - | - | 33% |
| **Bates, 2003** (n = 556) | 62 | 213 (38) | - | 8 | 556 (100) | 22***** |
| **Baxter, 1990** (n = 40) | - | 15 (38) | - | - | - | 35 |
| **Bernardi, 2008** (whole-leg CUS: n = 1,053) | 63 | 430 (41) | 0 | 7 | 430 (100) | 27***** |
| **Cavaye, 1990** (n = 53) | - | - | - | - | 53 (100) | 59 |
| **Chan, 2013** (n = 221) | 32 | 0 | - | - | 221 (100) | 7.7 |
| **Chance, 1991** (n = 70) | - | - | - | - | - | 20 |
| **Cornuz, 1999** (n = 997) | 53 | 345 (35) | - | - | - | 15 |
| **Cornuz, 2002** (n = 278) | 60 | 173 (62) | 50 (18) | - | 194 (70) | 30 |
| **Dybowska, 2015** (n = 1,048) | 61 | - | - | - | 1,048 (100) | 5.7 |
| **Elias, 2003** (n = 623) | - | - | - | - | 623 (100) | 33 |
| **Gibson, 2009** (n = 1,002) | 58 | 395 (39) | - | 7 | 940 (94) | **limited CUS:** 25 **whole-leg CUS:** 38 |
| **Gudmundsen, 1990** (n = 150) | 62 | 78 (52) | - | - | - | 40 |
| **Heijboer, 1992** (n = 83) | 60 | 35 (42) | - | 17 | 0 | 53 |
| **Kennedy, 1999** (n = 38) | 68 | 16 (42) | 5 (13) | - | 24 (63) | 42 |
| **Le Gal, 2012** (n = 210) | 33 | 0 | 26 (12) | - | 210 (100) | 12 |
| **Le Gal, 2006** (n = 162) | 29 | 0 | - | - | - | 19 |
| **Lensing, 1989** (n = 225) | 56 | 99 (44) | - | 9 | 225 (100) | 32 |
| **Linkins, 2013** (n = 1,723) | 62 | 640 (37) | - | - | 1542 (89) | 13***** |
| **Mantoni, 1989** (n = 90) | 53 | 46 (51) | - | - | - | 41 |
| **Mitsunaga, 2017** (n = 1,295) | 64 | 576 (44) | - | - | - | 9.4 |
| **Pasquariello, 1999** (n = 77) | 67 | - | - | - | - | 28 |
| **Prandoni, 2002** (n = 205) | 62 | 100 (49) | 205 (100) | - | - | 28 |
| **Quintavalla, 1992** (n = 165) | 68 | 74 (45) | - | - | 0 | 56 |
| **Rose, 1990** (n = 69) | 54 | 47 (68) | 19 (28) | - | 39 (57) | 45 |
| **Schutgens, 2003** (n = 827) | 59 | 309 (37) |  |  | 827 (100) | **single limited CUS:** 10 **serial limited CUS:** 52 |
| **Sluzewski, 1991** (n = 174) | 58 | 67 (39) | 0 |  | 118 (68) | 40 |
| **Stevens, 2004** (n = 445) | 56 | 145 (33) | 0 | 7 | - | 14 |
| **Stevens, 2013** (n = 183) | 53 | 63 (34) | 0 |  | 183 (100) | 9.3 |
| **Subramaniam, 2005** (n = 526) | 55 | 191 (36) | 26 (5) | - | 526 (100) | 22 |
| **Ten Wolde, 2002** (n = 1,739) | 61 | 651 (37) | 19 (1) | 7 | 1,739 (100) | **single limited CUS:** 2.8 **serial limited CUS:** 45 |
| **Tick, 2002** (n = 811) | 62 | 289 (36) | - | - | 811 (100) | 13***** |
| **Wells, 1997** (n = 593) | 57 | 249 (42) | 0 | 9 | 593 (100) | 17***** |
| **Wells, 1999** (n = 150) | 64 | 74 (49) | 0 | 6,6 | 0 | 20***** |
| **Wells, 2003** (n = 1,096) | 58 | 460 (42) | 202 (18) | 7,9 | 1,096 (100) | **single limited CUS:** 7.0 **serial limited CUS:** 28***** |
| **Wells, 1995** (n = 495) | - | - | 0 | - | 495 (100) | 22 |

Abbreviations: CUS: compression ultrasonography, DVT: deep vein thrombosis, PE: pulmonary embolism
* DVT prevalence in the subgroup of patients that was included in the meta-analysis.
