# Supplementary material for: Diagnostic accuracy of three ultrasonography strategies for deep vein thrombosis of the lower extremity: A systematic review and meta-analysis
Source: PLoS One. 2020 Feb 11;15(2):e0228788. doi: 10.1371/journal.pone.0228788 (PMC7012434; doi:10.1371/journal.pone.0228788)
Supplement: S7 Appendix — Abbreviations: CUS: compression ultrasonography PTP: pretest probability Patients were all-comers or were selected for CUS imaging with the use of a diagnostic algorithm consisting of a pretest probability (PTP) assessment and/or D-dimer. PTP was classified as either low, moderate, or high, or as ‘DVT likely’ or ‘DVT unlikely’. (DOCX) [file pone.0228788.s007.docx]

**S7 Appendix. Selection of patients prior to ultrasonography examination**

| **Single limited CUS** |  |
| --- | --- |
| Ageno, 2015 | PTP ‘DVT likely’ and negative D-dimer / PTP ‘DVT unlikely’ and positive D-dimer |
| Anderson, 2003 | Low PTP and positive D-dimer |
| Schutgens, 2003 | High PTP and negative D-dimer |
| Ten Wolde, 2002 | Negative D-dimer |
| Tick, 2002 | Low PTP |
| Wells, 2003 | PTP ‘DVT unlikely’ / PTP ‘DVT unlikely’ and positive D-dimer |
| **Serial limited CUS** |  |
| Anderson, 1999 | Moderate PTP |
| Bates, 2003 | Positive D-dimer |
| Gibson, 2009 | PTP ‘DVT unlikely’ and positive D-dimer / PTP ‘DVT likely’ |
| Linkins 2013 | Moderate PTP and positive D-dimer / high PTP |
| Prandoni, 2002 | All-comers |
| Schutgens, 2003 | Positive D-dimer |
| Sluzewski, 1991 | All-comers |
| Ten Wolde, 2002 | Positive D-dimer |
| Wells, 1997 | Moderate PTP |
| Wells, 1999 | Moderate PTP |
| Wells, 2003 | PTP ‘DVT likely’ |
| **Whole-leg CUS** |  |
| Ageno, 2015 | PTP ‘DVT likely’ and positive D-dimer |
| Bernardi, 2008 | All-comers |
| Cornuz, 2002 | All-comers |
| Gibson, 2009 | PTP ‘DVT unlikely’ and positive D-dimer / PTP ‘DVT likely’ |
| Stevens, 2004 | All-comers |
| Stevens, 2013 | PTP ‘DVT likely’ |
| Subramaniam, 2005 | All-comers |

Abbreviations: CUS: compression ultrasonography PTP: pretest probability

Patients were all-comers or were selected for CUS imaging with the use of a diagnostic algorithm consisting of a pretest probability (PTP) assessment and/or D-dimer. PTP was classified as either low, moderate, or high, or as ‘DVT likely’ or ‘DVT unlikely’.
