# Supplementary material for: Diagnostic accuracy of three ultrasonography strategies for deep vein thrombosis of the lower extremity: A systematic review and meta-analysis
Source: PLoS One. 2020 Feb 11;15(2):e0228788. doi: 10.1371/journal.pone.0228788 (PMC7012434; doi:10.1371/journal.pone.0228788)
Supplement: S8 Appendix — Abbreviations: CI: confidence interval, CUS: compression ultrasonography, DVT: deep vein thrombosis, PTP: pretest probability assessment *Patients undergoing CUS were either all-comers or were referred for imaging based on pretest probability assessment and/or D-dimer testing †Proximal limited CUS includes examination of the popliteal vein up to the femoral vein, extended limited CUS also includes the calf trifurcation. ‡P-value for meta-regressionSubgroup analyses for age, body mass index, history of venous thromboembolism, duration of symptoms, ultrasonography modalities, ultrasonography operator, and retrospective versus prospective study design were hampered as the number of studies in several subgroups was lower than 2. (DOCX) [file pone.0228788.s008.docx]

**S8 Appendix. Subgroup analyses - Summary estimates diagnostic accuracy of compression ultrasonography in studies that used clinical follow-up as a reference standard**

| **CUS technique** | **Overall** | **DVT prevalence** | | | **Patient selection*** | | | **Symptom duration** | | | **Publication date** | | | **Proximal vs extended limited CUS†** | | | |
| --- | --- | --- | --- | --- | --- | --- | --- | --- | --- | --- | --- | --- | --- | --- | --- | --- | --- |
|  |  | **≤ 15%** | **> 15%** | **P-value‡** | **All-comers** | **PTP and/or D-dimer** | **P-value‡** | **≤ 7 days** | **> 7 days** | **P-value‡** | **≤2003** | **>2003** | **P-value‡** | **Proximal** | **Extended** | **P-value‡** |  |
| **Single limited CUS** |  |  |  |  |  |  |  |  |  |  |  |  |  |  |  |  |  |
| Number of studies | 6 | - | - | - | - | - | - | - | - | - | - | - | - | - | - | - |  |
| Number of patients | 2,079 | - | - | - | - | - | - | - | - | - | - | - | - | - | - | - |  |
| Failure rate, % (95% CI) | 1.4  (0.83-2.5) | - | - | - | - | - | - | - | - | - | - | - | - | - | - | - |  |
| Proportion positive results, % (95% CI) | 6.4  (3.5-11) | - | - | - | - | - | - | - | - | - | - | - | - | - | - | - |  |
| **Serial limited CUS** |  |  |  |  |  |  |  |  |  |  |  |  |  |  |  |  |  |
| Number of studies | 11 | 2 | 9 | - | 2 | 9 | - | 3 | 2 | - | 9 | 2 | - | 2 | 9 | - |  |
| Number of patients | 3,360 | 471 | 2,889 | - | 379 | 2,981 | - | 443 | 446 | - | 2,737 | 623 | - | 379 | 2,981 | - |  |
| Failure rate, % (95% CI) | 1.9  (1.4-2.5) | 1.0  (0.4-2.6) | 1.8  (1.3-2.6) | 0.16 | 2.0  (0.65-5.8) | 1.8  (1.3-2.5) | 0.41 | 1.8  (0.8-4.2) | 1.6  (0.7-3.8) | 0.98 | 2.0  (1.4-2.8) | 1.4  (0.5-3.9) | 0.67 | 2.0  (0.6-5.8) | 1.8  (1.3-2.5) | 0.41 |  |
| Proportion positive results, % (95% CI) | 25  (18-34) | 12  (9.6-16) | 29  (21-37) | 0.03 | 32  (22-45) | 24  (16-33) | 0.50 | 19  (14-26) | 18  (14-23) | 0.79 | 27  (20-37) | 17  (8.3-31) | 0.21 | 32  (22-45) | 24  (16-33) | 0.50 |  |
| **Whole-leg CUS** |  |  |  |  |  |  |  |  |  |  |  |  |  |  |  |  |  |
| Number of studies | 7 | 2 | 5 | - | 4 | 3 | - | - | - | - | - | - | - | - | - | - |  |
| Number of patients | 3,159 | 628 | 2531 | - | 2,302 | 857 | - | - | - | - | - | - | - | - | - | - |  |
| Failure rate, % (95% CI) | 1.0  (0.6-1.6) | 0.7  (0.3-1.9) | 1.1  (0.7-1.9) | 0.43 | 0.91  (0.52-1.6) | 1.0  (0.4-2.6) | 0.87 | - | - | - | - | - | - | - | - | - |  |
| Proportion positive results, % (95% CI) | 25  (16-36) | 12  (7.4-18) | 32  (23-42) | 0.001 | 22  (16-30) | 28  (9.0-60) | 0.58 | - | - | - | - | - | - | - | - | - |  |

Abbreviations: CI: confidence interval, CUS: compression ultrasonography, DVT: deep vein thrombosis, PTP: pretest probability assessment

*Patients undergoing CUS were either all-comers or were referred for imaging based on pretest probability assessment and/or D-dimer testing

†Proximal limited CUS includes examination of the popliteal vein up to the femoral vein, extended limited CUS also includes the calf trifurcation.

‡P-value for meta-regression
Subgroup analyses for age, body mass index, history of venous thromboembolism, duration of symptoms, ultrasonography modalities, ultrasonography operator, and retrospective versus prospective study design were hampered as the number of studies in several subgroups was lower than 2.
