# Supplementary material for: Diagnostic accuracy of three ultrasonography strategies for deep vein thrombosis of the lower extremity: A systematic review and meta-analysis
Source: PLoS One. 2020 Feb 11;15(2):e0228788. doi: 10.1371/journal.pone.0228788 (PMC7012434; doi:10.1371/journal.pone.0228788)
Supplement: S9 Appendix — Abbreviations: CI: confidence interval, CUS: compression ultrasonography, DVT: deep vein thrombosis, PI: prediction interval * Tau-squared (τ2) represents the between-study variance and indicates the degree of heterogeneity. †The failure rate is the proportion of patients with a negative ultrasonography at baseline who were diagnosed with venous thromboembolism during follow-up. (DOCX) [file pone.0228788.s009.docx]

**S9 Appendix. Sensitivity analysis including all studies regardless of quality - Summary estimates diagnostic accuracy of compression ultrasonography in studies that used clinical follow-up as a reference standard**

| **Ultrasonography technique** | **Studies, n** | **Patients, n** | **DVT prevalence, median (IQR)** | **Proportion of positive results**  **(95% CI; 95% PI)** | **τ^2^*** | **Failure rate**†  **(95% CI; 95% PI)** | **τ^2^*** |
| --- | --- | --- | --- | --- | --- | --- | --- |
| Single limited CUS | 9 | 3,640 | 9.4 (6.8-13) | 8.1%  (4.8-13; 1.2-38) | 0.65 | 1.2%  (0.8-1.8; 0.6-2.5) | 0.52 |
| Serial limited CUS | 11 | 3,360 | 25% (18-34) | 25%  (18-33; 6.8-60) | 0.60 | 1.9%  (1.4-2.5; 1.1-3.2) | 0.51 |
| Whole-leg CUS | 12 | 6,151 | 21% (14-30) | 20%  (14-29; 4.1-60) | 0.65 | 0.9%  (0.6-1.3; 0.3-2.3) | 0.53 |

Abbreviations: CI: confidence interval, CUS: compression ultrasonography, DVT: deep vein thrombosis, PI: prediction interval

* Tau-squared (τ^2^) represents the between-study variance and indicates the degree of heterogeneity.
†The failure rate is the proportion of patients with a negative ultrasonography at baseline who were diagnosed with venous thromboembolism during follow-up.
