# Supplementary material for: Diagnostic accuracy of three ultrasonography strategies for deep vein thrombosis of the lower extremity: A systematic review and meta-analysis
Source: PLoS One. 2020 Feb 11;15(2):e0228788. doi: 10.1371/journal.pone.0228788 (PMC7012434; doi:10.1371/journal.pone.0228788)
Supplement: S10 Appendix — Abbreviations: DVT: deep vein thrombosis. (DOCX) [file pone.0228788.s010.docx]

**S10 Appendix. Distribution of proximal and distal deep vein thrombosis diagnosed with whole-leg compression ultrasonography in studies that used clinical follow-up as a reference standard**

| **Study** | **Proximal DVT, n (%)** | **Isolated distal DVT, n (%)** |
| --- | --- | --- |
| Ageno, 2015 | 118 (59) | 82 (41) |
| Bernardi, 2008 | 213 (77) | 65 (23) |
| Cornuz, 2002 | 49 (61) | 31 (39) |
| Gibson, 2009 | - | - |
| Stevens, 2004 | - | - |
| Stevens, 2013 | 6 (38) | 10 (62) |
| Subramaniam, 2005 | - | - |

Abbreviations: DVT: deep vein thrombosis
