# Supplementary material for: Diagnostic accuracy of three ultrasonography strategies for deep vein thrombosis of the lower extremity: A systematic review and meta-analysis
Source: PLoS One. 2020 Feb 11;15(2):e0228788. doi: 10.1371/journal.pone.0228788 (PMC7012434; doi:10.1371/journal.pone.0228788)
Supplement: S11 Appendix — Abbreviations: CI: confidence interval, CUS: compression ultrasonography, DVT: deep vein thrombosis, NPV: negative predictive value, PI: prediction interval, PPV: positive predictive value * Tau-squared (τ2) represents the between-study variance and indicates the degree of heterogeneity. †The false negative rate equals 1-NPV. (DOCX) [file pone.0228788.s011.docx]

**S11 Appendix. Sensitivity analysis including all studies regardless of quality - Summary estimates diagnostic accuracy of compression ultrasonography in studies that used contrast venography as a reference standard**

| **Ultrasonography technique** | **Studies, n** | **Patients, n** | **DVT prevalence, median (IQR)** | **Sensitivity**  **(95% CI; 95% PI)** | **τ^2^*** | **Specificity**  **(95% CI; 95% PI)** | **τ^2^*** | **PPV**  **(95% CI; 95% PI)** | **τ^2^*** | **NPV**  **(95% CI; 95% PI)** | **τ^2^*** | **False negative rate**†  **(95% CI; 95% PI)** |
| --- | --- | --- | --- | --- | --- | --- | --- | --- | --- | --- | --- | --- |
| Single limited CUS | 6 | 1,128 | 36% (25-41) | 96%  (90-98; 71-100) | 0.63 | 97%  (96-98; 95-98) | 0.50 | 95%  (91-97; 84-98) | 0.53 | 97%  (96-99; 95-99) | 0.51 | 2.5%  (1.5-4.2; 1.1-5.4) |
| Whole-leg CUS | 7 | 415 | 42% (34-49) | 83%  (71-91; 40-97) | 0.63 | 88%  (81-93; 66-97) | 0.56 | 85%  (78-89; 76-90) | 0.50 | 87%  (82-90; 80-91) | 0.50 | 13%  (9.6-19; 8.8-20) |

Abbreviations: CI: confidence interval, CUS: compression ultrasonography, DVT: deep vein thrombosis, NPV: negative predictive value, PI: prediction interval, PPV: positive predictive value
* Tau-squared (τ^2^) represents the between-study variance and indicates the degree of heterogeneity.

†The false negative rate equals 1-NPV
